# A longitudinal study of influenza A viral detection in bulk tank and pen-level milk collected from dairy farms in California affected by highly pathogenic avian influenza H5N1

**DOI:** 10.3168/jds.2025-27819

**Published:** 2026-05-04

**Authors:** C. Stenkamp-Strahm, B. Melody, P. Brinson, B. McCluskey, J. Lombard

**Affiliations:** 1 Department of Clinical Sciences, College of Veterinary Medicine and Biomedical Sciences, Colorado State University, Fort Collins, CO 80523-1678; 2 AgNext, College of Agricultural Sciences, Colorado State University, Fort Collins, CO 80521; 3 Lander Veterinary Clinic, Turlock, CA 95380

**Keywords:** influenza A virus, bulk tank milk, H5N1

## Abstract

Highly pathogenic avian influenza virus H5N1 has been infecting dairy herds in the United States since its initial incursion into cows in early 2024. Although national strategies have aimed to detect affected herds during this outbreak, information regarding longitudinal herd-level viral detection has not been formally studied. To help understand viral shedding of H5N1 in milk from H5N1-affected dairy herds over time, an outbreak investigation was conducted on dairy farms in California in the fall and winter of 2024. The primary objective of this observational investigation was to identify initial H5N1 infection in these California herds and then evaluate viral distribution via longitudinal bulk tank milk (BTM) testing. A secondary objective was to evaluate viral movement via longitudinal pen-level milk (PLM) sampling in a subset of affected herds. Daily BTM samples were submitted from 19 originally noninfected herds that were clients of a single veterinary clinic and volunteered to participate in this study. Samples were tested for influenza A (IAV) via real-time reverse transcription (rRT)-PCR. In a subset of 5 herds, daily PLM IAV testing was completed soon after initial BTM IAV detection. Overall, a total of 4,749 BTM and 2,922 PLM samples were submitted for testing, and all herds eventually became infected with IAV. After initial detection, daily detections of IAV occurred in BTM for a minimum of 33 d, with some herds continuing to have detection at the end of the sampling period (>75 d). The BTM cycle threshold nadirs were seen 1 to 3 wk after initial detection. In PLM-tested herds, virus was detected in samples from all pens of cattle soon after BTM detection and, in some cases, was seen in all pens on the first day of PLM sampling. Overall, these results support BTM testing as an effective early detection tool for dairy H5N1 outbreaks but suggest that surveillance strategies that do not test aggregate milk from all lactating cows on an operation or have a long interval between BTM testing days may fail to identify all affected herds. Future investigations should study farm-level factors associated with long-term viral detection in some herds and assess detection sensitivities of the IAV rRT-PCR assay used on aggregate milk samples.

## INTRODUCTION

Highly pathogenic avian influenza virus of the H5N1 subtype has been infecting dairy cattle and spreading among dairy herds in the United States since early 2024 (Nguyen et al., 2025). Before this outbreak, influenza A viruses (**IAV**) of the H5N1 subtype (hereafter **H5N1**) had infected wild birds, poultry, and other mammals and humans but had not shown evidence of efficient mammal-to-mammal transmission (Gilbertson and Subbarao, 2023; Tammiranta et al., 2023; Graziosi et al., 2024). Since the start of this outbreak, H5N1 has demonstrated efficient transmission within and between herds and has infected 1,088 dairy herds in 19 states as of early March 2026 (USDA, 2026). Transmission of the H5N1 virus among dairy farms and between individual dairy cows remains poorly understood, however. Routes of H5N1 exposure in dairy cattle may include oral, respiratory, or teat end exposure via milking equipment (Baker et al., 2025; Campbell et al., 2026; Halwe et al., 2025). Cattle that are infected with H5N1 may show signs of clinical disease that vary in type and severity, with high H5N1 viral loads often detected in their milk. Milk shedding of virus may persist for several weeks, and virus has also been found in other cattle excretions, including nasal discharge and urine (Caserta et al., 2024; Halwe et al., 2025; Baker et al., 2025). Of note, although the B3.13 genotype of H5N1 clade 2.3.4.4b remains the predominant genotype clinically affecting dairy herds in the US, an additional genotype (D1.1) was also detected in herds in early and late 2025 (AVMA, 2025; USDA, 2025a). This genotype purportedly causes milder signs of clinical disease in cows (K. Sarlo Davila, M. Wymore Brand, E. Putz, P. Boggiatto, H. Seger [all of USDA Agricultural Research Service {**ARS**}, National Animal Disease Center {**NADC**}, Ames, IA; A. Baker, Tavis Anderson [both of USDA ARS, Ames, IA]; C. Hutter, A. Buckley [both of USDA ARS, NADC, Ames, IA]; A. Vander Plaats, R. Friedrich, P. Gordon [all of Iowa State University, Ames, IA]; B. Arruda, C. Souza, B. Davidson, M. Palmer [all of USDA ARS, NADC, Ames, IA]; unpublished data).

Dairy herd bulk tank milk (**BTM**) samples represent pooled milk from lactating cows on an operation and have historically been used for pathogen surveillance (Nobrega et al., 2023a,b). Although the milk from sick cows is diverted from the saleable stream, H5N1 has been detected in BTM samples from herds before and after the onset of H5N1 clinical signs (NMPF, 2024; Spackman et al., 2024; USDA, 2025b; Stenkamp-Strahm et al., 2026). Ideal detection techniques for H5N1 on dairy operations are still being refined, but modeling of BTM testing done in the week before clinical signs reported by farm personnel has shown an almost 70% probability of detecting an IAV-positive herd (USDA, 2025b). In the spring of 2024, the USDA initially attempted to reduce interstate spread of the virus by implementing a federal order mandating that lactating cows have their milk tested for IAV before interstate movement (USDA, 2024a). To date, testing for virus in BTM or silo milk samples has also been used for initial detection, quarantine, or continuous monitoring of H5N-affected herds at both state (CDA, 2024; CDFA, 2024; UDAF, 2024) and federal (USDA, 2024a,b) levels.

During these H5N1 surveillance activities, aggregate milk testing of affected herds has occurred on either a weekly, monthly, or more infrequent basis, with methodology of sample collection varying across states. The best methods for H5N1 surveillance in dairy cattle are unknown, in part because the patterns of herd-level H5N1 detections over time are not understood. To determine the patterns and persistence of BTM detections on H5N1-affected dairy operations, daily testing of aggregate milk samples taken throughout the outbreak in each herd is necessary, and this would include samples taken in the period before clinical disease detection in cows. Knowledge of these BTM detection patterns may reveal an ideal methodology for continued nationwide surveillance of H5N1. It would also serve to inform dairies that have not yet experienced H5N1 infection regarding the timeframe for disease management and quarantine should they become infected in the future.

Given that we lack complete understanding of dairy H5N1 transmission, once a herd is affected by H5N1, it is possible that individual pens of cattle are exposed to the virus at different rates or by different routes (or both). To the authors’ knowledge, milk testing for H5N1 at the pen level (pen-level milk; hereafter denoted as **PLM**) has not been previously performed in dairy herds affected during this outbreak. Pen-level milk testing done after initial H5N1 detection is made in BTM may inform within-herd H5N1 movement patterns and viral transmission within a dairy farm.

In the summer of 2024, California had not yet detected any dairy cases of H5N1, with the first infected farm reported on August 30, 2024. The virus initially emerged in the Central Valley region of the state and subsequently spread, resulting in more than 770 affected farms by March, 2026 (USDA, 2026). In partnership with Lander Veterinary Clinic, dairies that had not yet exhibited clinical signs or tested positive for H5N1 were enrolled in this investigation in the fall of 2024. The presence of H5N1 in the region, combined with the availability of herds that were clinically unaffected, provided a unique opportunity to evaluate herds before and throughout their H5N1 outbreak periods.

The primary objective of this investigation was to identify initial H5N1 infection in these California herds and then evaluate viral distribution via longitudinal bulk tank milk testing. A secondary objective was to evaluate viral movement via longitudinal pen-level milk sampling on a subset of these dairies.

## MATERIALS AND METHODS

### Design of Investigation

The San Joaquin and Sacramento Valleys represent the southern and northern portions of California’s dairy-dense Central Valley, respectively, and collectively have over 1.5 million milking cows (USDA NASS, 2024). Lander Veterinary Clinic (**LVC**) is a large livestock practice located in Turlock, California, near the center of the Central Valley. The LVC clinicians serve ~150 dairy operations composed of ~250,000 lactating cows located throughout the Central Valley. The LVC requested participation from 25 dairy operations that were active clients of the clinic, represented the broad location and breadth of operations the clinic services, and were logistically able to support daily bulk tank milk sampling. All LVC-serviced operations, including those recruited, had not experienced H5N1 infection at the time of recruitment. Sampling for this study was conducted by convenience, and all operation participation was voluntary, therefore no sample size calculation was performed. Of herds successfully recruited for the BTM testing, a convenience sample of 5 herds were asked to participate in PLM testing. Overall, 19 operations agreed to participate in this repeated cross-sectional investigation that included 2 overlapping sampling schemes to detect IAV: longitudinal BTM testing (n = 19) and longitudinal PLM testing (n = 5). All herds were enrolled between September 10, 2024, and November 14, 2024, with sample submissions beginning after personnel training at the specific dates for each farm listed in Supplemental Table S1 (see Notes).

### Sample Collection and Processing

For BTM samples, milk haulers were instructed to submit one aggregate milk sample from each tank that was transferred to the tanker for shipping, following standard BTM collection procedures. Each individual bulk tank sample may or may not have represented all lactating cows on the operation, and no farms in this investigation used direct loads (i.e., no farms lacked refrigerated bulk tank storage). Samples were kept at −18°C until courier pick-up and then driven to LVC on ice and kept refrigerated until testing. Herds submitted between 1 and 8 unique BTM samples per day, depending on the number of shipments, operation size, and daily milk production. Each operation submitted daily BTM samples starting at enrollment and continued until February 12, 2025. A single farm stopped daily submissions after January 10, 2025, given their earlier outbreak timeframe and lack of detection in submitted samples for the prior 3 wk.

For PLM samples, on a subset of herds (n = 5), dairy staff were trained by LVC personnel to collect daily milk samples from each pen (n = 6–13) of lactating cows that contributed to the saleable bulk tank(s). The PLM sample collection began 6 to 18 d after initial BTM detection of IAV. Pooled, nonsaleable milk from hospital or other pens was not sampled. A QualiTru Sampling Systems peristaltic pump (Part No. 500200) with a sanitary fitting, a sterile 7-channel septum, and sterile collection bags was installed by LVC personnel to collect milk just before bulk tank deposit or just after the receiver jar, as deemed appropriate based on each farm’s milking system. Standard operating procedures for collection were followed, and between each milking pen a new sterile septa channel and fluid line were fitted, and residual milk was flushed from the receiver jar(s) several times to allow it to clear before subsequent collection. Samples were kept at −18°C until courier pick-up and then driven to LVC on ice and kept refrigerated until testing. Once at LVC laboratory, BTM and PLM samples were tested for IAV via real-time reverse transcription (**rRT**)-PCR assay within 24 h of collection. The LVC followed personal protective equipment standards used by the California Animal Health and Food Safety laboratory and American Association of Veterinary Laboratory Diagnosticians member laboratories with gloves, N95 masks, face shields, and safety glasses. Briefly, RNA extraction was completed using 50 μL of sample diluted in 150 μL PBS with a MagMax CORE Nucleic Acid Purification Kit (Applied Biosystems, Cat. A32700) paired with a Swine Influenza Virus RNA Test Kit (Applied Biosystems, Cat. 4415200) on a KingFisher Flex 96 System (Thermo Fisher). A QuantStudio 6 or QuantStudio 5 Pro Real-Time PCR System (Thermo Fisher, Cat. A43168, A28569) was used according to manufacturer’s instructions to perform rRT-PCR for IAV. Reactions were performed using 8 μL of extracted RNA from each sample. A cycle threshold (**Ct**) cut-off value of 40 was used. Samples were stored at 4°C after testing and then bulk shipped to Iowa State University (**ISU**) for parallel testing. The ISU Veterinary Diagnostic Laboratory, in Ames, IA, is a National Animal Health Laboratory Network (**NAHLN**) laboratory. At ISU NAHLN, sample RNA was isolated, and rRT-PCR assays for IAV were performed per NAHLN standard operating procedures and protocols, using a cycle threshold cut-off value of 40. The Ct values <40 were considered positive. Annually, each NAHLN laboratory is required to successfully complete National Veterinary Services Laboratories (**NVSL**)-administered proficiency tests and quality control procedures for identification of IAV.

For this study, each herd was considered an experimental unit, and each collected bulk tank or pen-level milk sample was considered an observational unit. At study outset, no power calculations were conducted for detecting IAV in aggregate milk from enrolled operations, as data regarding the average number of bulk tanks shipped on each farm was not formally collected, and this was not specifically tracked during the duration of the study. Further, test sensitivity and specificity of the IAV PCR, at the time of the study, had not been formally determined for raw aggregate milk samples.

After initial detection of IAV in the BTM from each operation, each herd was confirmed to be infected with IAV H5N1 clade 2.3.4.4b genotype B3.13 via testing at NVSL in Ames, Iowa.

### Data Cleaning, Validation, and Analysis

In the case of LVC, rRT-PCR Ct results were sent directly from the laboratory, whereas ISU NAHLN results were reported to the USDA via the laboratory messaging system, and then these results were aggregated weekly via USDA and sent to CSU. The BTM and PLM results were cleaned via manual inspection of dates and submission IDs, validated, and merged for each farm. The herd date of initial clinical signs and designation of cattle types in each pen were determined via herd-level records and discussion with veterinarians and herd management personnel. The date of initial clinical signs was determined based on observing any number of the following clinical signs in cows on farms with BTM detection: a drop in milk production, a drop in rumination, mastitis, fever, lethargy, or nasal discharge.

The BTM samples on some farms were not collected every day due to weekend or holiday dates where sample collection was not possible. Additionally, some samples were only tested at LVC because they were misplaced or lost in transit to the ISU NAHLN laboratory, and study funding sources also necessitated that some samples be tested only at LVC. Overall, 7% (545/7671) of rRT-PCR results used in the analysis were from testing done at LVC (Supplemental Table S1), whereas all other rRT-PCR results used in analyses were from testing at the ISU NAHLN laboratory. Although defining laboratory accuracy was not a goal of the current work, rRT-PCR Ct values of samples measured at both laboratories were compared to ensure equivalence. A correlation of Ct values from LVC and ISU was completed by selecting dates from 5 farms with low (<25), medium (25–35), and high (>35) Ct values measured at each testing laboratory. Pearson correlation and Wilcoxon signed rank for paired sample tests were performed using these values, and a correlation regression plot was created (Supplemental Figure S1, see Notes). Samples between the 2 laboratories were highly correlated (Pearson = 0.954) and lacked significant difference (Wilcoxon signed rank = 0.796).

After data validation, the number of days from BTM detection to the onset of clinical signs in cows, the number of days from BTM detection or onset of clinical signs to the lowest achieved BTM Ct value, and the number of days from BTM detection to the first day all samples submitted were nondetected, were calculated. The lowest BTM and PLM Ct value measured for each farm over the course of the study was determined, and the lowest daily Ct values measured for BTM and PLM were graphically depicted over time. The lowest Ct value from each pen was described by the time from initial BTM detection and the onset of clinical signs in cows. There were some days that farms submitted BTM samples with detection and also submitted BTM samples that were nondetected. The number of these days was quantified. A Fisher’s exact test was used to test the null hypothesis that breed, parlor type, and times milked per day did not vary between herds enrolled for BTM or PLM testing. A Wilcoxon rank sum test was used to test the null hypothesis that herd size did not vary between herds enrolled for BTM or PLM testing. All statistical and descriptive analyses were performed using R (version 2024.12.1+563 or later; R Core Team; https://www.R-project.org/).

## RESULTS

Of the 19 operations enrolled, a majority had more than 1,000 lactating cows (73.6% for the BTM study, 100% for the PLM study; Table 1). About half of operations milked their lactating cows 3 times per day (57.8% for the BTM study, 40% for the PLM study). For the BTM study, 68.4% of the operations were comprised of primarily Holstein cows, whereas 10.5% had primarily Jersey cows, and the other 21.1% of farms were composed of multiple breeds or mixed breeds. For the subset of farms that submitted PLM samples, 60.0% of operations were comprised of primarily Holsteins, whereas the rest were comprised of primarily Jersey cows. Operations enrolled across studies had several different parlor types, including herringbone, parallel, rotary, robotic, and flat barn (Table 1). Of herds that submitted BTM samples, the highest percentage had herringbone parlors (42.1% compared with 21.1%, 26.3%, 5.3%, and 5.3% for parallel, robotic, rotary, and flat barn parlors, respectively), whereas an equal number of herds that submitted PLM samples had herringbone and rotary parlors (40% for herringbone and rotary, compared with 20% parallel, respectively). The number of times cows were milked per day differed between BTM- and PLM-tested herds (Fisher’s exact test *P* = 0.035), but parlor type, breed, and herd size were not significantly different between these groups (*P* > 0.05 for all comparisons). Farm size, parlor type, breeds, and classes of cattle represented on each enrolled operation are described in Supplemental Table S2, see Notes.

**Figure 1.**
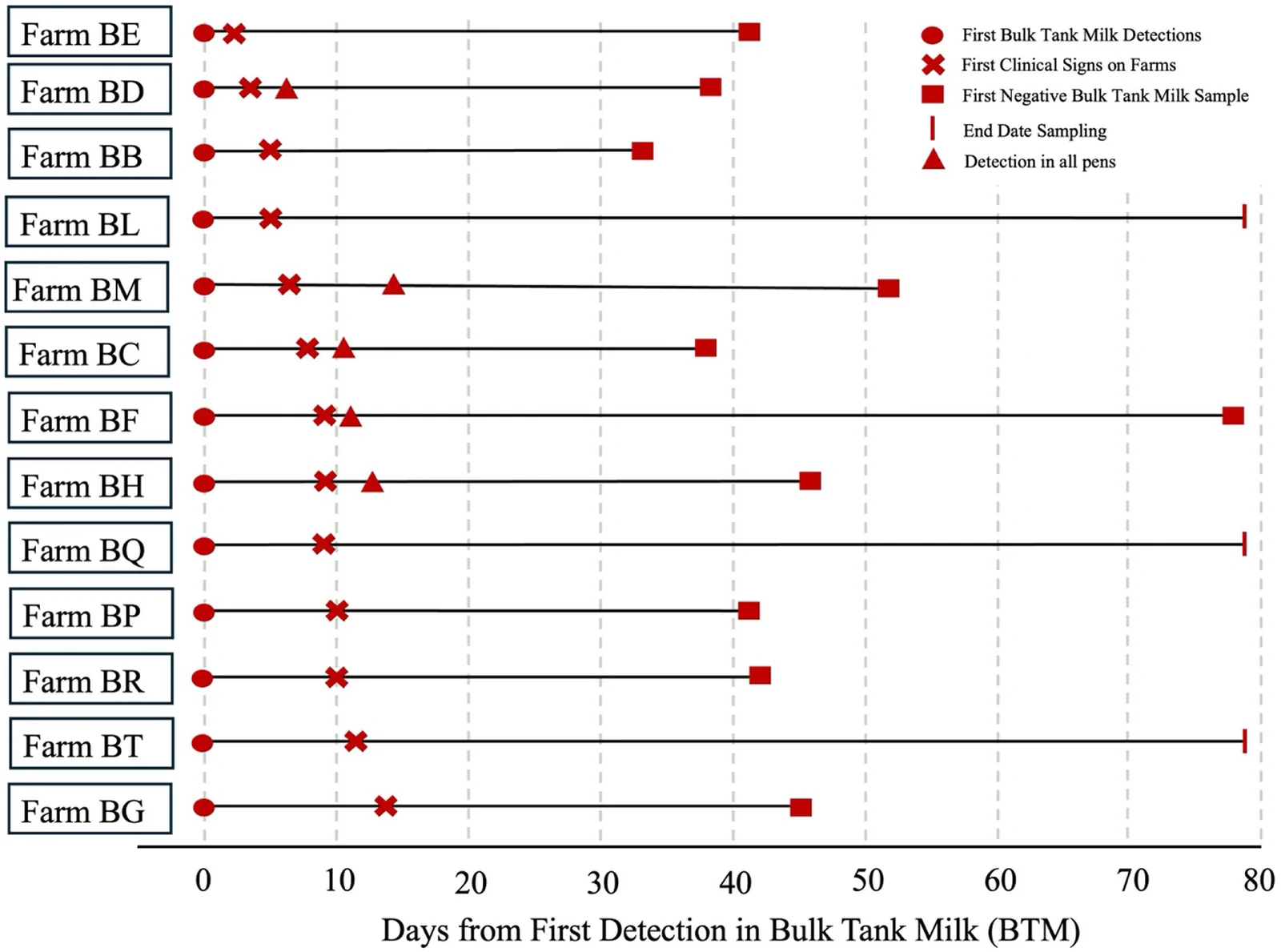
Timeline showing the number of days from BTM influenza A virus (IAV) detection to clinical signs in 13 California dairy herds, IAV detection in PLM from all pens (for farms enrolled in the PLM study), and date of either the first day with all negative tanks or end date of sampling. Study herds shown had consistent daily submissions of BTM throughout the study period.

During the study period, a total of 7,671 aggregate milk samples were submitted from enrolled operations; 4,749 were BTM and 2,922 were PLM. The total number of BTM and PLM samples submitted by each farm can be found in Supplemental Table S2. A total of 16 farms submitted BTM samples consistently throughout the study period. Three farms (BO, BU, and BW) were omitted from subsequent analyses because daily sample submissions were either missing or inconsistently provided, so downstream analyses included data from the 16 other farms (Table 2). Additionally, 3 farms (BI, BN, and BS) were missing BTM sample submissions in the days leading up to first detection. These farms were omitted from calculations that assessed first positive date to the date with lowest Ct, the date of clinical signs in cows, and the first date with all negative tanks (Table 2, Figure 1).

### Bulk Tank Milk Detection

Each enrolled farm had initial BTM IAV detection between October 24, 2024, and December 11, 2024, which was 15 to 56 d after initial sampling. The number of days from initial IAV detection to the first day when all BTM samples were negative ranged from 33 to 77 d (4–7 wk), with 3 farms having continuous daily detections until the end of the study period (Table 2, Figure 1). Figure 1 provides a timeline for 13 farms that had complete data, showing initial BTM detection, first clinical signs of H5N1 in cows, the first date that all samples submitted from each farm after detection were negative, or the end date of sampling, or both.

**Figure 2.**
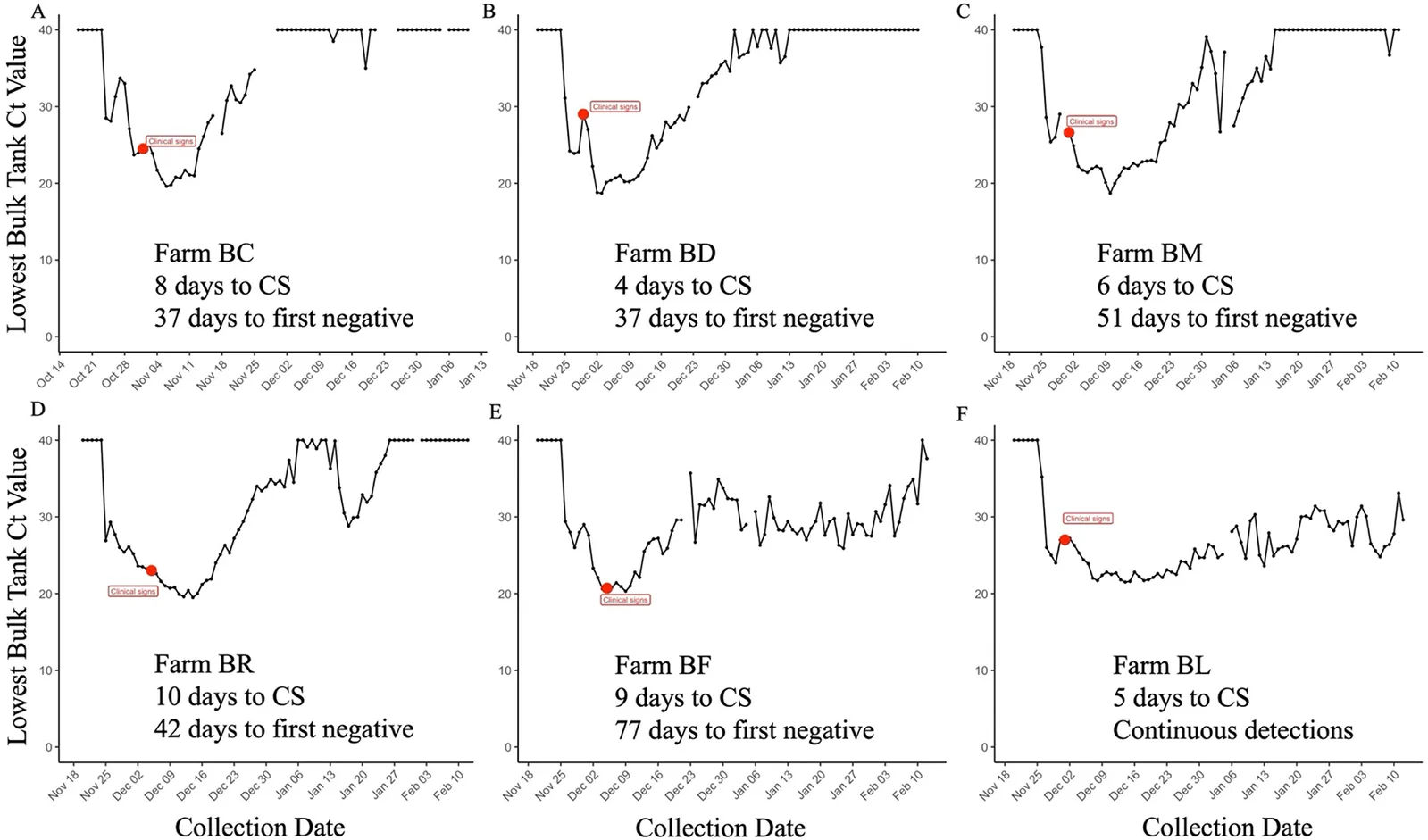
Panels A–F: Lowest daily influenza A (IAV) Ct values from bulk tank milk (BTM) samples submitted from 6 California dairy farms over time. These farms had a different number of days from BTM IAV detection to when cows experienced clinical signs (CS), and different periods of time (37–77 d) between initial IAV detection to when all tank samples submitted on a day were negative. Two of the farms represented (Panels E and F) continued to have BTM IAV detections to the end of the study period.

The lowest BTM Ct value achieved during the outbreak period for enrolled farms was between 18.7 and 21.6 and occurred from 8 to 23 d after initial BTM detection (Table 2). Each farm experienced clinical signs of H5N1 disease in cows between 3 and 14 d after initial BTM detection, and clinical signs occurred one to 14 d before each farm’s lowest achieved BTM Ct (Table 2). Curves of the lowest daily BTM Ct values over time for 6 representative farms can be seen in Figure 2, with curves from the additional 10 study farms found in Supplemental Figure S2, see Notes. Of note, 13 farms experienced days where, on the same day of sampling, both detected and nondetected IAV BTM results occurred (range in number of days with detection and nondetection = 0–19; Supplemental Table S1).

### Pen-Level Milk Detection

**Figure 3.**
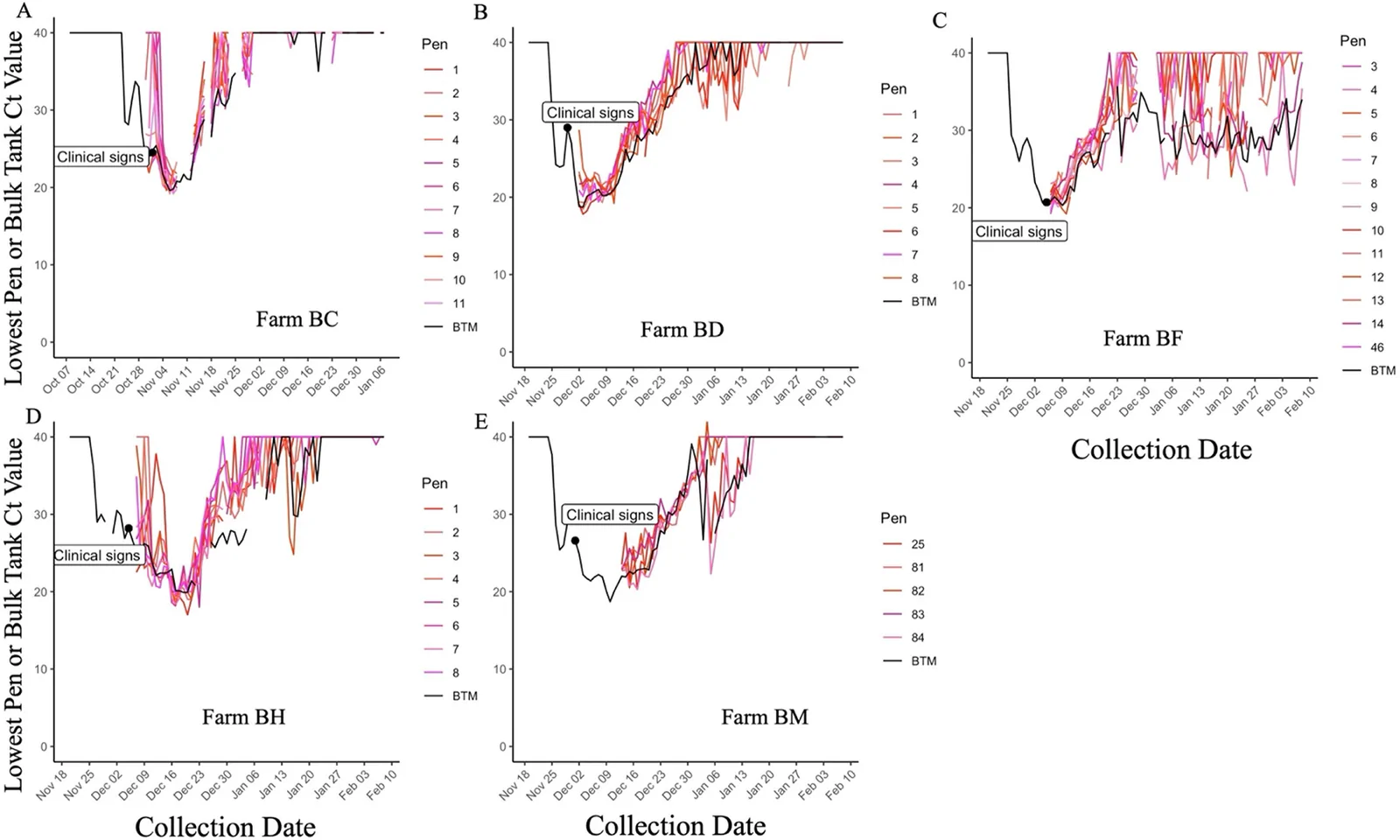
Panels A–E: Lowest daily influenza A (IAV) Ct values from bulk tank milk or pen-level milk samples submitted from 5 California dairy farms enrolled in the pen-level detection study, over time. Some farms (Panels B, C, and E) had detection in all pens on the first day pen-level samples were submitted. One farm (Panel C) had detections in BTM and some pen-level samples to the end of the study period. One farm (Panel B) had several days where detections were made in pen-level milk samples even though BTM had become test-negative.

The PLM samples were collected on 5 enrolled farms starting 6 to 18 d after each farm had initial BTM IAV detection. Brief descriptions of the cattle types present in each sampled pen can be found in Supplemental Table S3, see Notes. The IAV was detected in the milk from all pens on these operations 7 to 18 d after initial BTM detection, although 3 of 5 farms had detection in all pens on their first pen-level sample collection date (at 7-, 10-, and 18-d post BTM detection, respectively; Table 2, Figure 1). The number of days from clinical signs to IAV detection in all PLM samples ranged from 1 to 12 d for these farms (Table 2, Figure 1), although these estimates could be long given the delay in starting PLM sampling. Curves showing daily PLM Ct values with daily BTM Ct values from each of the 5 sampled operations are seen in Figure 3. The PLM samples collected near the time of the BTM Ct nadir on each farm tended to have Ct values similar to those of the main bulk tank (Figure 3). Of note, there were between 0 and 14 sample days when PLM IAV detections were made, whereas BTM samples from the same herd were test-negative for IAV (Table 2, Figures 3B and 3D). A single farm that submitted PLM samples continued to have BTM detections until the end of the study period (Figure 3C). In the final days of sampling, only 2 of the 6 pens sampled appeared to be contributing to continued detections on this farm. Cattle in these pens included cows that had recently calved and early lactation cows between their first and fourth lactation (Supplemental Table S3, see Notes).

## DISCUSSION

This study details daily BTM IAV detections on dairies affected by the H5N1 virus. Because herds were enrolled before initial detection of IAV, trends in viral load (Ct values) measured from the beginning to the end of the outbreak period on each dairy could be analyzed and visualized over time, giving estimates to the timeframe for H5N1 outbreak management.

Trends could be seen in the IAV detection timelines seen on most study dairies. In general, the time from first detection to the day when all BTM samples were test-negative occurred between 4 and 7 wk. Some farms (4/16; 25%) did not fit this trend, however, and had consistent daily detections of IAV lasting >75 d. The BTM detections with a similarly prolonged time course have been seen in prior work looking at affected Colorado farms (USDA, 2025b). Reasons for prolonged IAV BTM detection on any dairy may be related to maintenance of the virus in certain farm environments, movement of naïve cows onto the operation, exposure of naïve cattle on the farm, or various approaches to the management/quarantine of sick cattle that may continue to shed infectious virus. A single herd with prolonged detection (farm BF) also participated in the PLM study and had detections in just 2 pens of cattle at the end of the study period. This operation sends heifers offsite at 5 to 6 mo of age to be bred and raised until just before calving. One of the pens with prolonged detection was composed of cows that had recently calved, suggesting that potentially naïve cattle coming back to the operation may play a role in prolonged BTM detection.

The previous finding that IAV was present in BTM before reported clinical signs in cattle (NMPF, 2024) was confirmed in this study. Farms enrolled were made aware of their BTM detection status and likely anticipated that they would have clinically ill cattle shortly after detection. However, clinical signs of H5N1 were seen anywhere from 3 to 14 d after initial detection on participating operations. Although the definition of H5N1 clinical signs may have differed across farms, these findings reinforce that BTM testing is an effective method for early virus detection on dairies. Furthermore, the Ct nadir for BTM samples occurred 1 to 14 d after the presence of reported clinical signs. Therefore, even after sick cows are likely removed from the saleable milk string, there is still an increase in viral load within the BTM. This is likely due to either unidentified clinical cows or nonclinical cows shedding viral RNA into their milk.

The PLM sampling results require cautious interpretation, as the small number of participating farms limits the generalizability of findings. For farms that submitted PLM samples, the H5N1 virus was detected in all pens within a relatively short time window (1–12 d) of clinical signs being detected in the herd. This timeframe is likely an overestimation, however, as the virus was detected in all sampled pens on 3 of 5 enrolled farms on the first date of PLM sampling. Regardless, these results suggest rapid within-herd transmission, although the route of viral spread remains unknown. Results from intramammary infection experiments initially suggested contaminated milking equipment as a primary mode of H5N1 transmission in dairy cows (Baker et al., 2025; Halwe et al., 2025), but infection using these methods has not been successfully reproduced in a laboratory setting (Lee et al., 2026). Although other dairy pathogens can be spread via contaminated milking equipment, history suggests that cow-to-cow transmission via this route in conventional milking systems may be relatively inefficient (Baxter et al., 1992; Deng et al., 2021) and unlikely to result in the proficient movement of virus observed. In addition to parlor surfaces and milking equipment, work by our group and others has shown H5N1 viral detection in dairy bioaerosols, wastewater, and the personal protective equipment of dairy personnel (Campbell et al., 2026; Stenkamp-Strahm et al., 2026), and these entities may also play a role in H5N1 cattle transmission.

When PLM was collected and tested near the peak of the outbreak (lowest achieved BTM Ct), Ct values from pens were often similar to those of the BTM measured on the same day, and measured nadirs were similar between samples from PLM and BTM. A single farm in the study (farm BC) submitted PLM samples before the date of herd clinical signs. On this farm, virus was detected in >30% of pens on the day clinical signs were first reported. From a management perspective, this suggests that by the time clinical observations and segregation of sick cows are possible, the virus may have already spread considerably within a herd.

On the 4 farms enrolled for PLM sampling that did not have continuous detections, there were between 4 and 14 sample days when PLM IAV detections were made, but BTM from the same herd had become test-negative for IAV. Additionally, 13 farms experienced days where they submitted multiple BTM samples, with some having IAV detection, whereas others were nondetected. Herds without these differing test results in a single day were herds that frequently submitted single daily samples (data not shown). These test result discrepancies are likely due to varying contributions of milk from different pens of cattle into the bulk tank, or each bulk tank not necessarily representing the milk from all lactating cows on the farm, or both. This may also be due to detection sensitivities of the IAV rRT-PCR assay used on pooled milk.

Results from BTM and PLM detections in this study may be used to guide a discussion of dairy H5N1 surveillance. For example, some herds may have outbreak windows that are less than or very close to 1 mo in duration. Monthly or more infrequent antigen-only testing may show false-negative results in herds that experience short outbreak windows, given the timeframes seen in this investigation. If using BTM for herd-level detection surveillance or for quarantine release, there is a need to ensure samples that represent all milking cows on an operation (i.e., samples representing all milk shipped on a given day) are tested for the highest detection sensitivity. Further, in this study there were days when pen-level samples from a herd had IAV detection, whereas BTM samples from the same day were test-negative. This was observed toward the end of herd outbreak periods and when test results were trending toward nondetection. This supports a notion that the sensitivity of BTM IAV testing decreases as viral load decreases (USDA, 2025b), but at the time of this writing, very little has been published regarding the detection sensitivity of IAV in aggregate PLM samples. Future work should focus on measuring IAV assay detection sensitivity in raw milk, especially in cases where only a few animals may be contributing low levels of viral RNA to an aggregated sample.

### Limitations

Enrollment of herds in this investigation was done by convenience, so the representativeness of this data is unknown. Enrolled farms were instructed to submit a single milk sample from each bulk tank shipped daily. This was in an attempt to represent all lactating cows on an operation, because cows may be milked multiple times and milk from multiple bulk tanks may be shipped each day, depending on farm size. The number of samples submitted compared with the number of bulk tanks shipped was not monitored, however, so the authors are limited in knowing if all cows on each operation were truly represented in Ct measures, or whether duplicate samples from shipped tanks were submitted. Pen-level milk samples were not collected until 7 to 18 d after initial viral detection due to equipment and personnel constraints and due to the time required for virus confirmation. The time from viral detection in the bulk tank to time of viral detection in all pens is therefore likely to be an underestimation. We also cannot extrapolate viral loads in the milk from a given tank or pen of animals to how many individual cows may be shedding virus. This limits our understanding as to the exact speed of intraherd viral spread; although virus was detected in all pens relatively soon after detection in the BTM, it is possible that only a few individuals were contributing viral RNA to collected samples. Moreover, the number of times cows were milked each day on operations enrolled for PLM sampling was different than that of operations enrolled for strictly BTM sampling. The small sample size of the PLM study limits external validity, making it uncertain if balancing this characteristic would have appreciably changed the results. The work described was only performed in herds with the B3.13 genotype and done in one state (CA), and comparing the magnitude of each individual herd’s outbreak is not possible given BTM Ct values alone. Although standard curves are not routinely completed by NAHLN laboratories on samples submitted for outbreak testing, a Ct nadir value of 18 (the lower end of the range seen in this work) would likely equate to ~1,900,000 viral copies of RNA, whereas a Ct value of 21 (the upper end of the range seen in this work) would equate to ~228,000 copies (S. Robbe-Austerman, NVSL, Ames, IA, personal communication). Finally, there is currently no standardized case definition for H5N1 disease in dairy cattle. As a result, the criteria for a producer to consider a cow to be “clinical” for H5N1 vary across affected farms, and the reported date of first clinical signs may have been influenced by producers’ awareness that the virus was already present in each herd enrolled in this investigation.

## CONCLUSIONS

Our findings describe IAV detection trends and variabilities in aggregate milk samples taken from dairy herds affected by H5N1 over time. Viral loads tend to peak within 3 wk of initial IAV detection and generally decline by 2 mo. Some herds did not fit these trends, however, which highlights a need to investigate farm-level factors associated with prolonged IAV detection. Although this work has not clarified transmission routes of the virus between cows, PLM detections suggest that intraherd spread of the virus is relatively quick, especially considering the clinical disease onset in each herd. Our results support aggregate milk testing as an effective early detection tool for dairy H5N1. Surveillance may be strengthened if testing occurs more frequently than once per month, and aggregate milk samples that represent all lactating cows on each operation are used. The authors also recommend completing studies that focus on the detection sensitivity of the IAV rRT-PCR assay when used on aggregate milk samples.

## NOTES

Data used in this publication was made possible, in part, by an agreement from the USDA’s APHIS VS (APHIS VS; Cooperative Agreement 25-9419-0731) and the National Institute of Allergy and Infectious Diseases (Rockville, MD), National Institutes of Health, Department of Health and Human Services, under Contract No. 75N93021C00016. This publication may not necessarily express the views of APHIS VS. Support was provided by the California Department of Food and Agriculture (CDFA; Sacramento, CA). Any opinions, findings, conclusions, or recommendations expressed in this publication are those of the author(s) and do not necessarily reflect the view of the CDFA. The authors thank the following personnel of Lander Veterinary Clinic (Turlock, CA) for their work collecting and characterizing samples: Yvette Guzman, Ernesto Padilla, Alondra Ramirez, Carina Montañez, Jessica Pacheco, and Giselle Leon. We thank Barb Petersen at Sunrise Veterinary Services for her expertise and coordination with this study and Natalie Urie (Colorado State University, Fort Collins, CO) for her work in reviewing and editing the manuscript. Additionally, we thank the Iowa State University Veterinary Diagnostic Laboratory (Ames, IA) team for their testing of aggregate milk samples. We also thank the California Department of Food and Agriculture for their collaboration and cooperation during this work. Artificial intelligence (AI) was not used in the writing of any section of the manuscript body. During the preparation of this work, the authors used ChatGPT to generate the interpretive summary and reviewed and edited the content. The authors take full responsibility for that section of the manuscript. Supplemental material for this article is available at https://doi.org/10.17605/OSF.IO/TFJ87. The Colorado State University Institutional Animal Care and Use Committee (IACUC) determined this study to be exempt from IACUC approval. CSU IACUC #5980. The authors have not stated any conflicts of interest.

## Figures and Tables

**Table 1.** Demographic characteristics of 19 California dairy herds enrolled in H5N1 detection studies, by study participation

		BTM detection study		PLM detection study	
Characteristic	Level	Number	Percent	Number	Percent

	Total	19	100.0	5	100.0
Herd size	<1,000	5	26.3	0	0.0
	1,000– ≤2,000	7	36.8	3	60.0
	>2,000	7	36.8	2	40.0
Times milked per day	≤2	4	21.1	1	20.0
	2–3^1^	4	21.1	2	40.0
	≥3	11	57.8	2	40.0
Breed	Holstein ≥95%	13	68.4	3	60.0
	Jersey ≥95%	2	10.5	2	40.0
	Mixed	4	21.1	0	0.0
Parlor type	Herringbone	8	42.1	2	40.0
	Parallel	4	21.1	1	20.0
	Rotary	5	26.3	2	40.0
	Robotic	1	5.3	0	0.0
	Flat barn	1	5.3	0	0.0

1 Farm will milk cattle either 2 or 3 times per day, depending on production.

**Table 2.** Bulk tank milk and pen-level milk longitudinal detection results by herd

Herd	No. days from detection to a day with all negative tanks	Lowest BTM Ct value	No. days from detection to lowest BTM Ct value	No. days from BTM detection to CS	No. days from CS to lowest BTM Ct value	No. days from BTM detection to PLM sampling	No. days from BTM detection to detection in all pens	No. days from CS to detection in all pens	No. days with PLM detection and nondetection in BTM

BB	33	19.6	11	5	6				
BC	37	19.6	13	8	5	6	11	3	4
BD	37	18.7	8	4	4	7	7^2^	3^2^	14
BE	41	19.2	13	3	10				
BF^1^	77	20.3	13	9	4	10	10^2^	l^2^	0
BG	35	19.6	15	14	1				
BH	46	19.9	23	9	14	11	15	6	6
BI^3^		20.9			8				
BL^1^	>77	21.5	18	5	13				
BM	51	18.7	15	6	9	18	18^2^	12^2^	4
BN^3^		19.5			4				
BP	41	20.3	15	10	5				
BQ^1^	>77	21.6	21	9	12				
BR	42	19.5	19	10	9				
BS^3^		19.3			3				
BT^1^	>77	19.4	17	11	6				
All herds range	33–≥77	18.7–21.6	8–23	3–14	1–14	6–18	7–18	1–12	

1 Continued to have BTM detections to the end of study period.

2 Detections were made in all pens on the first day of pen-level sampling.

3 Sample submissions in days before initial detection were missing, making accurate calculation of days from detection to the first negative sampling day and lowest Ct value impossible.

## Nonstandard abbreviations used

ARS: Agricultural Research Service
BTM: bulk tank milk
CS: clinical signs of H5N1 disease in cows
Ct: cycle threshold
H5N1: highly pathogenic avian influenza virus of the H5N1 subtype
IAV: influenza A virus
ISU: Iowa State University
LVC: Lander Veterinary Clinic
NADC: National Animal Disease Center
NAHLN: National Animal Health Laboratory Network
NVSL: National Veterinary Services Laboratories
PLM: pen-level milk
rRT: real-time reverse transcription
